# CYP98A monooxygenases: a key enzyme family in plant phenolic compound biosynthesis

**DOI:** 10.1093/hr/uhaf074

**Published:** 2025-03-10

**Authors:** Zheng Zhou, Yonghao Duan, Yajing Li, Pan Zhang, Qing Li, Luyao Yu, Cuicui Han, Juncheng Huo, Wansheng Chen, Ying Xiao

**Affiliations:** State Key Laboratory of Discovery and Utilization of Functional Components in Traditional Chinese Medicine, Institute of Chinese Materia Medica, Shanghai University of Traditional Chinese Medicine, 1200 Cailun Road, Pudong New Area, Shanghai 201203, China; Navy Special Medical Centre, Second Military Medical University, 800 Xiangyin Road, Yangpu District, Shanghai 200433, China; State Key Laboratory of Discovery and Utilization of Functional Components in Traditional Chinese Medicine, Institute of Chinese Materia Medica, Shanghai University of Traditional Chinese Medicine, 1200 Cailun Road, Pudong New Area, Shanghai 201203, China; State Key Laboratory of Discovery and Utilization of Functional Components in Traditional Chinese Medicine, Institute of Chinese Materia Medica, Shanghai University of Traditional Chinese Medicine, 1200 Cailun Road, Pudong New Area, Shanghai 201203, China; State Key Laboratory of Discovery and Utilization of Functional Components in Traditional Chinese Medicine, Institute of Chinese Materia Medica, Shanghai University of Traditional Chinese Medicine, 1200 Cailun Road, Pudong New Area, Shanghai 201203, China; Department of Pharmacy, Changzheng Hospital, Second Military Medical University, 415 Fengyang Road, Huangpu District, Shanghai 200003, China; Navy Special Medical Centre, Second Military Medical University, 800 Xiangyin Road, Yangpu District, Shanghai 200433, China; Navy Special Medical Centre, Second Military Medical University, 800 Xiangyin Road, Yangpu District, Shanghai 200433, China; Department of Pharmacy, Changzheng Hospital, Second Military Medical University, 415 Fengyang Road, Huangpu District, Shanghai 200003, China; State Key Laboratory of Discovery and Utilization of Functional Components in Traditional Chinese Medicine, Institute of Chinese Materia Medica, Shanghai University of Traditional Chinese Medicine, 1200 Cailun Road, Pudong New Area, Shanghai 201203, China; Department of Pharmacy, Changzheng Hospital, Second Military Medical University, 415 Fengyang Road, Huangpu District, Shanghai 200003, China; State Key Laboratory of Discovery and Utilization of Functional Components in Traditional Chinese Medicine, Institute of Chinese Materia Medica, Shanghai University of Traditional Chinese Medicine, 1200 Cailun Road, Pudong New Area, Shanghai 201203, China

## Abstract

Phenolic compounds are derived from the phenylpropanoid metabolic pathways of plants and include phenylpropionic acids, lignins, coumarins, and flavonoids. These compounds are among the most abundant and diverse classes of secondary metabolites found throughout the plant kingdom. Phenolic compounds play critical roles in the growth, development, and stress resistance of horticultural plants. Moreover, some phenolic compounds exhibit substantial biological activities, and they are widely utilized across various sectors, such as the pharmaceutical and food industries. The cytochrome P450 monooxygenase 98A subfamily (CYP98A) is involved mainly in the biosynthesis of phenolic compounds, mediating the *meta*-hydroxylation of aromatic rings in the common phenylpropane biosynthesis pathways of phenolic compounds. However, research on this family of oxidases is currently fragmented, and a systematic and comprehensive review has not yet been conducted. This review offers an exhaustive summary of the molecular features of the CYP98A family and the functions of CYP98A monooxygenases in the biosynthesis of different types of phenolic compounds. In addition, this study provides a reference for the exploration and functional study of plant CYP98A family enzymes. An enhanced understanding of CYP98A monooxygenases can help in the cultivation of high-quality horticultural plants with increased resistance to biotic and abiotic stresses and enhanced accumulation of natural bioactive compounds via metabolic engineering strategies. Moreover, the structural optimization and modification of CYP98A monooxygenases can provide additional potential targets for synthetic biology, enabling the efficient *in vitro* production of important phenolic compounds to address production supply conflicts.

## Introduction

**Figure 1:**
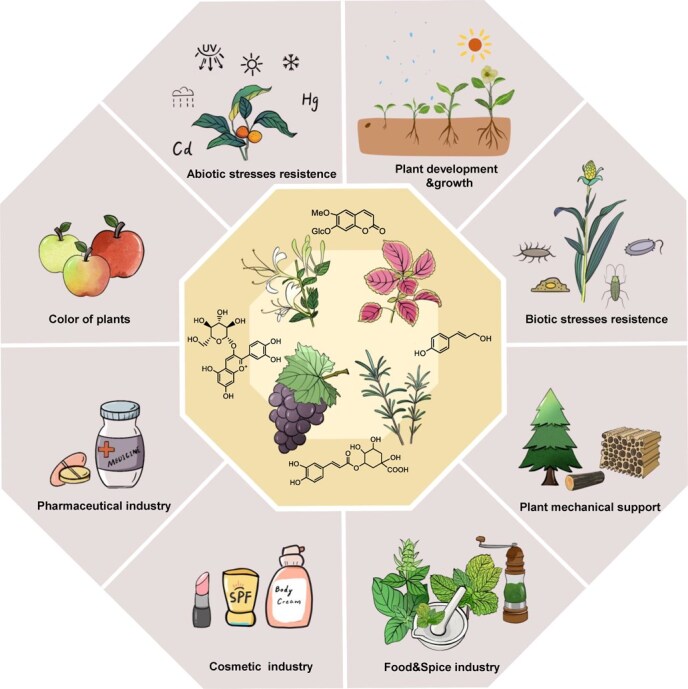
Functions of plant phenolic compounds. Phenolic compounds play critical roles in the growth and development of horticultural plants; the defence against biotic and abiotic stresses; the formation of plant pigments; the provision of mechanical support; and applications in the medical, cosmetic, and food industries.

Phenolic compounds, including phenylpropanoids (such as phenylpropionic acids, lignins, and coumarins) and flavonoids, are significant natural products of the secondary metabolites of plants. The structural characteristics of these compounds involve the presence of one or more hydroxyl groups within an aromatic ring. Phenolic compounds possess significant antioxidant and anti-inflammatory activities, along with anti-infection, antiviral, antibacterial, antiallergic, antihemorrhagic, and immune-boosting properties. Moreover, phenolic compounds play critical roles in horticultural plants and their ecological environments [1]. For example, under abiotic stresses, such as drought, salinity, and ultraviolet radiation, the phenylpropanoid biosynthetic pathway is activated, leading to increased production of phenolic acids in horticultural plants and thus helping them scavenge excessive amounts of reactive oxygen species (ROS) [2–4]. Certain compounds, such as coumarins and flavonoids, display notable resistance against plant pathogens, and they function as signalling molecules in the plant’s response to biotic stimuli [5–7]. In addition, lignin deposition provides mechanical support and enhances the lodging resistance of horticultural plants [8, 9]. Consequently, the biosynthesis and metabolic regulation of phenolic compounds have attracted substantial attention worldwide (Fig. 1).

**Figure 2:**
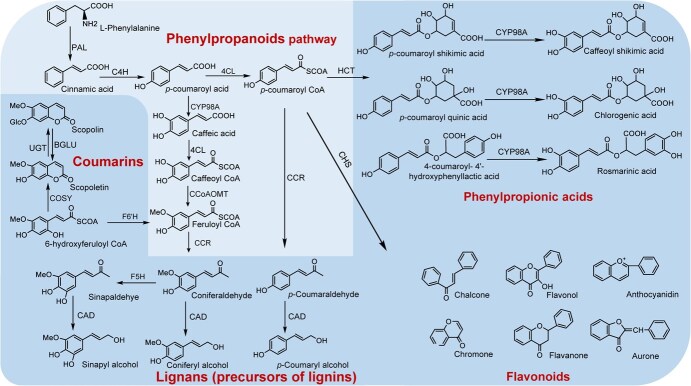
Biosynthetic pathways of plant phenolic compounds involving a series of reactions. L-Phenylalanine is converted into the key precursors *p*-coumaric acid and *p*-cinnamoyl-CoA, which are the starting points of important metabolic branches that are subsequently catalysed to produce various types of phenolic compounds, including phenylpropionic acid derivatives, coumarins, lignins, and flavonoids.

Although phenolic compounds have diverse biosynthetic pathways, phenylalanine generated via the shikimate pathway in plants acts as a universal precursor for the biosynthesis of phenolic compounds [10]. Phenylalanine participates in a series of enzymatic reactions within the phenylpropanoid pathway, culminating in the production of several precursor substances for phenolic compounds, including *p*-coumaric acid and *p*-coumaroyl-CoA. *p*-Coumaric acid undergoes *meta*-hydroxylation and acylation and can be converted into caffeic acid and caffeoyl-CoA. Under the action of caffeoyl-CoA *O*-methyltransferase (CCoAOMT), the phenolic compound is subsequently transformed into feruloyl-CoA. This product is further processed into coumarin compounds (specifically, scopoletin and its derivatives), and this process is facilitated by the actions of feruloyl-CoA-6′-hydroxylase (F6′H) and coumarin synthase (COSY). Feruloyl-CoA and coumaroyl-CoA can be transformed into three types of lignin monomers via the enzymatic action of cinnamoyl-CoA reductase (CCR) and cinnamyl alcohol dehydrogenase (CAD). Coumaroyl-CoA can interact with simple aromatic acids (such as shikimic acid, quinic acid, and 4-hydroxybenzoic acid) through the catalysis of BAHD acyltransferases, thereby producing phenylpropionic acid compounds. Alternatively, coumaroyl-CoA may undergo polymerization with malonyl-CoA, yielding flavonoid compounds through the action of chalcone synthase (CHS) (Fig. 2).

The plant cytochrome P450 monooxygenase 98A subfamily (CYP98A) is involved primarily in the *meta*-hydroxylation reactions of phenylpropanoid compounds. This family is referred to as *p*-coumarate 3-hydroxylase (C3H) because of its initial ability to catalyse the conversion of *p*-coumarate derivatives into corresponding caffeic acid derivatives. To date, extensive research has been conducted on the role of CYP98A in the biosynthetic pathways of phenolic compounds, covering aspects such as natural product biosynthesis, metabolic regulation, plant growth and development, and environmental stress responses [11–14]. However, a comprehensive overview summarizing and organizing research on this monooxygenase family that includes prospects for future studies on the functions of these enzymes is lacking. The aims of this study are to provide a review of the basic structure, classification, and biological functions of CYP98A monooxygenases and to offer additional references and insights for future studies.

## Characteristics of CYP and CYP98A monooxygenases

### Structural features of CYPs

CYP98A monooxygenases belong to the plant cytochrome P450 (CYP) family. CYP is a family of heme-containing monooxygenases that can undergo self-oxidation [15]. These enzymes are characterized by their use of haem as a cofactor, in which an iron atom is coordinated with a porphyrin ring. The cofactors are linked to a highly conserved heme-binding structural domain, symbolized as FXXGX_b_XXCXG, where X_b_ represents a cysteine residue with unique spectral properties. Cytochrome P450 derives its name from the fact that the cysteine complex that forms when CYP binds to reduced CO has the highest absorbance at 450 nm [16, 17].

### Classification methods and naming schemes for plant CYPs

CYPs are ubiquitously found across various life forms in nature, with the most extensive presence being in plants. Over 200 000 plant CYPs have been catalogued in public databases, accounting for approximately 1% of the total number of genes in the genome [18]. These proteins constitute the largest and most crucial family of enzyme proteins that participate in diverse life processes in plants.

CYPs have a clear naming scheme based on the similarity of amino acid sequences for their classification that divides the enzymes into three levels: families, subfamilies, and individual enzymes. Amino acid sequences with greater than 40% similarity are classified into the same family, whereas those with similarity levels between 40% and 55% are assigned to different subfamilies within the same family, as denoted by uppercase letters. Sequences with over 55% similarity belong to the same subfamily but are considered different individual enzymes, with Arabic numerals denoting the order in which these enzymes were identified. Terrestrial plant CYPs are segregated into families numbered 51, 71–99, 701–999, and greater than 7001 [19]. On the basis of evolutionary relationships, plant CYPs are classified into 11 clans, with the smallest family in each clan being designated as the name of the clan. The 11 clans are as follows: CYP51, CYP74, CYP97, CYP710, CYP711, CYP727, CYP746, CYP71, CYP72, CYP85, and CYP8611 [18]. The CYP71 family encompasses more than 50% of the CYPs in higher-level plants and plays a key role in plant secondary metabolism. For example, the CYP71 family significantly contributes to the biosynthesis of various sesquiterpenes, including capsidiol, artemisinin, and caryophyllene [20–23]. The CYP76 and CYP701 families are involved primarily in diterpene biosynthesis and include compounds such as tanshinone, gibberellin, Taxol, and abietate [24, 25]. The P450 genes in the CYP73A subfamily, such as cinnamate 4-hydroxylase, and those in the CYP93 family, such as flavone synthases, contribute to the formation of a flavonoid skeleton [26, 27]. The CYP706X and CYP82D subfamilies, which include C6 and C8 flavonoid hydroxylases, are essential for the biosynthesis of specific compounds, such as scutellarin, baicalin, and wogonin [28, 29]. P450 enzymes from the CYP80, CYP82, and CYP719 subfamilies are typically required for the biosynthesis of various alkaloids, including magnoflorine, morphine, sanguinarine, and noscapine [30–33]. CYP98 primarily participates in the *meta*-hydroxylation of phenylpropanoid compounds, consequently influencing the accumulation of phenolic compounds in plants [34, 35].

### Catalytic mechanisms of CYP and CYP98A enzymes

**Figure 3:**
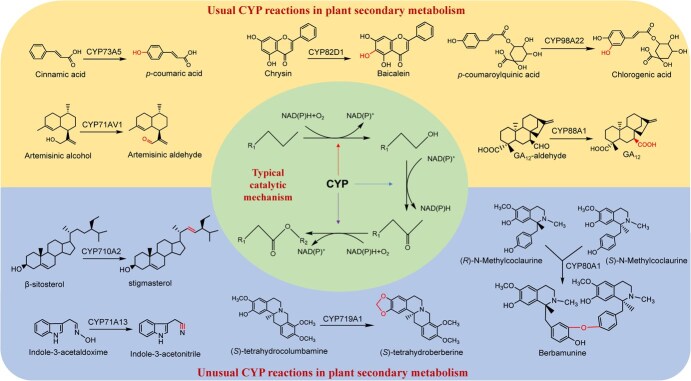
Catalytic mechanisms and reaction types catalysed by plant CYP monooxygenases.The upper panel refers to typical CYP reactions,whereas the lower panel depicts unusual CYP reactions in plant secondary metabolism.

The typical catalytic reaction facilitated by CYPs is predominantly associated with the monooxygenation of carbon atoms. In plants, the monooxygenase system found in the endoplasmic reticulum of eukaryotes contains two integral membrane proteins, namely, cytochrome P450 and NADPH-cytochrome P450 reductase (CPR), which contain the prosthetic groups FAD and FMN. When redox reactions occur, electrons are transferred from NAD(P)H to P450 via CPR, which coincides with the reduction of another oxygen molecule to form water [36]. This reaction can be generally delineated by the following equation: R + NAD(P)H + O_2_ + H^+^ → RO + NAD(P) + H_2_O. The activation of oxygen molecules, as mediated by CYPs, can involve many metabolic reactions, including hydroxylation, epoxidation, dehydrogenation, isomerization, C–C bond cleavage, dehalogenation, deamination, and heteroatom oxidation, culminating in the production of alcohols, aldehydes, ketones, carboxylic acids, and epoxides (Fig. 3).

### Subcellular locations of CYP and CYP98A enzymes

CYPs constitute a sizable class of conserved membrane proteins that are typically linked to the membranes of various organelles, including the endoplasmic reticulum, plastids, mitochondria, and the Golgi apparatus, where they metabolize a myriad of endogenous and exogenous compounds [37]. *Rg*CYP98A22 from *Ruta graveolens* and *Sm*CYP98A14 and *Sm*CYP98A75 from *Salvia miltiorrhiza* are all localized to the endoplasmic reticulum, where they associate with the membrane of the organelle to perform *meta*-hydroxylase functions [38, 39].

## Evolution of CYP98A

**Figure 4:**
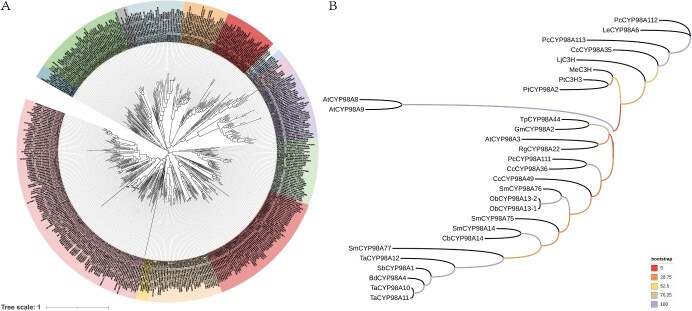
Phylogenetic distributions of the CYP71 clan and CYP98A family monooxygenases. The analysis was performed using the maximum likelihood method with MEGA10 software, and the results were polished with the iTOL tool. The protein data were downloaded from the National Center for Biotechnology Information (https://www.ncbi.nlm.nih.gov/). The accession numbers of each protein are presented in the figure.

CYPs are generally believed to have a polyphyletic origin, and they are primarily split into two fundamental branches: A-type and non-A-type [40]. A-type CYPs play a significant role in plant-specific metabolism and are responsible for the biosynthesis of various natural products [41]. Compared to A-type CYPs, non-A-type CYPs contain several more divergent lineages, showing a larger sequence resemblance to P450s in bacteria and animals; in addition, these CYPs function in lipid or hormone metabolism [42]. The CYP71 clan is the largest group of P450 enzymes in plants, encompassing families 71, 73, 75, 76, 77, 78, 79, 80, 81, 82, 84, 93, 98, 701, 703, 706, 709, 712, and 719, which are further divided into multiple subfamilies, exhibiting a vast array of functions, including plant secondary metabolism, defence, and adaptation to environmental stress. Most members of clan CYP71 are involved in the metabolism of aromatic and aliphatic amino acid derivatives, such as phenylpropanoids, indolic derivatives, glucosinolates, and cyanogenic glucosides, of small isoprenoids, such as mono- and sesquiterpenoids, triterpenoid derivatives, alkaloids, fatty acids, and hormone precursors, including GAs, and of potentially undiscovered novel hormones (Fig. 4A). Evidence from evolutionary studies reveals that although CYP98A genes are broadly distributed in terrestrial plants, their existence as single copies has been observed only in bryophytes, lycophytes, and ferns. Events, such as independent gene duplications and deletions, have been observed exclusively in angiosperms. Therefore, CYP98A genes are presumed to be present in the genomes of nearly all higher-level (vascular) plants [34, 35, 43]. An evolutionary analysis of the CYP98A genes has revealed that most members can be represented as single entities, except for four in *S. miltiorrhiza* and three each in *A. thaliana* and *Triticum aestivum*. Additionally, family members with similar catalytic properties form one cluster (Fig. 4B).

## Progress in functional studies of the role of CYP98A monooxygenases in phenolic compound biosynthesis

Since *At*CYP98A3 was first reported in *Arabidopsis thaliana* in 2001, a total of 29 additional CYP98A monooxygenases have been identified over the past 24 years [34]. Most of these genes catalyse the *meta*-hydroxylation reactions of 4-hydroxycinnamic acid esters, and they play key roles in important physiological and biochemical processes, such as secondary metabolite synthesis, three-dimensional growth, and pollen wall formation [12, 34, 38, 44, 45].

### Progress in research on the role of CYP98A monooxygenases in the biosynthesis of phenylpropionic acid natural products

Phenylpropionic acid natural products, or simple phenylpropanoids, constitute a broad class of phenylpropanoid compounds characterized by a ubiquitous C_6_–C_3_ skeleton. The basic structure is composed of a hydroxyl-substituted aromatic ring and an acrylic acid moiety, including phenylpropionic acids and phenyl lactic acids, such as cinnamic acid, coumaric acid, caffeic acid, rosmarinic acid (RA), caffeoyl shikimate, *p*-coumaroyl quinate, chlorogenic acid (caffeoyl quinate), and 4-hydroxyphenylacetic acid. These natural products exhibit remarkable biological activities and thus have extensive applications in different areas, such as the medicine, food, fragrance, and cosmetic fields.

Phenylpropionic acid compounds are derivatives of phenylalanine and tyrosine, which are produced via the shikimic acid pathway. Following catalytic modifications by a variety of enzymes, phenylalanine yields the crucial *p*-coumaroyl-CoA and is subsequently catalysed by members of BAHD acyltransferase families, resulting in the formation of various structurally complex and bioactive simple phenylpropanoid natural products. CYP98A enzymes participate in the *meta*-hydroxylation of the phenolic ring in a phenylpropionic acid structure, thereby playing a significant role in the biosynthesis of several key phenylpropionic acid compounds, including caffeoyl shikimate, chlorogenic acid, RA, caffeic acid, and danshensu (DSS).

#### Function of CYP98A in the biosynthesis of chlorogenic acid and caffeoyl shikimic acid

The role of CYP98A was initially identified in the biosynthesis of caffeoyl shikimic acid and chlorogenic acid. Both caffeoyl shikimic acid and chlorogenic acid are essential natural products in the plant phenylpropanoid biosynthetic pathway and are found in almost all higher plants, particularly in horticultural species of the Caprifoliaceae, Asteraceae, and Rosaceae families [46–48]. These compounds exhibit significant antioxidant and anti-inflammatory properties [49]. Research suggests that caffeoyl shikimic acid and chlorogenic acid are generated via a *meta*-hydroxylation reaction catalysed by CYP98A monooxygenases.

Most CYP98A monooxygenases derived from angiosperms utilize *p*-coumaroyl shikimate and *p*-coumaroyl quinate as preferred substrates, which catalyse the generation of caffeoyl shikimic acid and chlorogenic acid. However, substantial variations in the substrate selectivity and catalytic efficiency of these enzymes have been observed among different species. For example, *At*CYP98A3 from *A. thaliana* has a *meta*-hydroxylation capacity for *p*-coumaroyl shikimate that is fourfold greater than that for *p*-coumaroyl quinate [34]. *Ob*CYP98A13 from *Ocimum basilicum* has a *meta*-hydroxylation capacity for *p*-coumaroyl shikimate that is 5 to 10 times greater than that for *p*-coumaroyl quinate [50]. *Cc*CYP98A49 from *Cynara cardunculus* has a greater affinity for *p*-coumaroyl shikimate than for *p*-coumaroyl quinate [51]. Conversely, *Rg*CYP98A22 from *R. graveolens* metabolizes *p*-coumaroyl quinate to chlorogenic acid more efficiently than *p*-coumaroyl shikimate [39].

Additionally, in the same species, CYP98A monooxygenases exhibit significant differences in catalytic efficiency for different substrates. For example, *Pt*CYP98A23 and *Pt*CYP98A27 from *Populus trichocarpa* display comparable conversion rates for *p*-coumaroyl shikimate. However, *Pt*CYP98A23 results in a higher conversion rate for *p*-coumaroyl quinate than *Pt*CYP98A27 [35]. *Bd*CYP98A4 from *Brachypodium distachyon* prefers *p*-coumaroyl quinate and *p*-coumaroyl shikimate as substrates, and it actively catalyzes the synthesis of 4-coumaroyl benzoyl octanediamine [35]. *Cc*CYP98A35 and *Cc*CYP98A36 from *Coffea canephora* display distinct substrate selectivities and catalytic efficiencies. *Cc*CYP98A35 can catalyse the formation of caffeoyl shikimic acid and chlorogenic acid from *p*-coumaroyl shikimate and *p*-coumaroyl quinate at equivalent rates, whereas *Cc*CYP98A36 is solely capable of hydroxylating *p*-coumaroyl shikimate to produce caffeoyl shikimic acid [52]. Various studies have indicated that specific CYP98A monooxygenases selectively utilize either *p*-coumaroyl shikimate or *p*-coumaroyl quinate as substrates. For example, *Lj*C3′H from *Lonicera japonica*, *Tp*CYP98A44 from *Trifolium pratense*, *La*C3′H from *Leucojum aestivum*, and *Pc*CYP98A111 from *Phacelia campanularia* exhibit a preference for *p*-coumaroyl shikimate over *p*-coumaroyl quinate as their substrate [53–55]. The catalytic efficiency of *Ta*CYP98A10 in *T. aestivum* for *p*-caffeoyl shikimic acid is less than one-fifteenth that of *Ta*CYP98A11, and its efficiency for *p*-coumaroyl quinate is even lower. Only 11 amino acid disparities exist between the protein sequences of the two *Ta*CYP98A monooxygenases. Further investigation has revealed that the different catalytic preferences of *Ta*CYP98A10 and *Ta*CYP98A11 for substrates are attributable to the distinct orientation of the Arg 51 residue [56].

Additionally, CYP98A monooxygenases can influence horticultural plant growth, development, and stress resistance by affecting the biosynthesis and metabolism of phenylpropionic acid compounds, thereby increasing the antioxidant capacity of a plant. Research has revealed that even trace amounts of selenium can upregulate the expression of the CYP98A gene, which results in the substantial accumulation of chlorogenic acid in apple roots. This accumulation consequently impacts the equilibrium between plant growth and defence mechanisms [57]. CYP98A is a key gene involved in the response of *Hemerocallis citrina* to infection with *Puccinia hemerocallidis*, and it is highly expressed in resistant strains via an increase in lignin content [58]. Upon ultraviolet radiation exposure, the expression levels of *Le*CYP98A6, *Lj*C3′H, and *Tp*CYP98A44 increase, and this process is accompanied by the increased accumulation of secondary metabolites, such as chlorogenic acid and caffeoyl shikimic acid, suggesting that the CYP98A gene plays a critical role in the biosynthesis of phenylpropionic acid compounds [51, 53, 59]. Moreover, the CYP98A gene has been identified as a key gene involved in the biosynthetic pathway of chlorogenic acid in peaches, with a positive correlation between the expression levels of the CYP98A gene and the content of chlorogenic acid in peach fruits [60]. When horticultural plants are subjected to biotic and abiotic stresses, the expression of the CYP98A enzyme is significantly upregulated, and this process is accompanied by an increase in phenolic compounds, such as chlorogenic acid and caffeoyl shikimic acid. These phenolic compounds can scavenge ROS or act as signalling molecules, and their accumulation enhances plant resistance to external stresses, such as UV radiation and pathogens. Therefore, by increasing the expression of CYP98A, it is possible to further increase the levels of phenolic compounds, thereby improving the resistance of horticultural plants to environmental stresses.

#### Function of CYP98A monooxygenases in the biosynthesis of salvianolic acids

Salvianolic acids such as DSS, caffeic acid, and RA present substantial antioxidant properties and the ability to scavenge free radicals. These compounds exhibit an array of potential pharmacological activities, including anti-hepatic fibrosis, anti-cardiovascular disease, and neuroprotective capabilities, thus rendering them invaluable for various applications [61–63]. Salvianolic acids are prevalent in numerous plant species (specifically, Lamiaceae and Boraginaceae plants). These plants are used not only as ornamental plants for landscaping but also as traditional medicinal plants. CYP98A monooxygenases primarily mediate *meta*-hydroxylation reactions in the biosynthesis of salvianolic acids, catalysing the formation of caffeic acid, DSS, and RA.

For caffeic acid biosynthesis, two hypotheses are considered. The first hypothesis is that the biosynthesis of caffeic acid from *p*-coumaric acid is facilitated by CYP98A monooxygenases. *At*CYP98A3 from *A. thaliana* and *Pt*CYP98A27 from *P. trichocarpa* can successfully hydroxylate free *p*-coumaric acid, leading to the production of caffeic acid [35, 64]. Conversely, emerging research has proposed an alternative mechanism, suggesting that the transformation of *p*-coumaric acid to caffeic acid is not mediated by CYP98A monooxygenases. The soluble enzyme ascorbate peroxidase/4-coumarate 3-hydroxylase (APX/C3H) is present in various plants, including *A. thaliana*, *Sorghum bicolor,* and *B. distachyon*. *In vitro* catalytic assays have shown the ability of this enzyme to directly hydroxylate free *p*-coumaric acid to caffeic acid [65]. However, this theory has been refuted by the studies of Bixia Zhang and Vahid Karimzadegan, suggesting that *S. bicolor* APX/C3H polymerizes *p*-coumaric acid into a novel, unidentified product rather than directly hydroxylating it to caffeic acid [66, 67].

Most studies on the involvement of CYP98A in the biosynthesis of salvianolic acids have focused predominantly on the biosynthesis of RA. Many scholars have reported the participation of CYP98A monooxygenases in RA biosynthesis in various horticultural plant species, including Lamiaceae plants, such as *Coleus blumei* and *S. miltiorrhiza*, and Boraginaceae plants, such as *Lithospermum erythrorhizon* and *P. campanularia*.

Eberle *et al.* initially isolated *Cb*CYP98A14, an enzyme capable of catalysing the 3-hydroxylation of 4-coumaroyl-3′,4′-dihydroxyphenyllactic acid and the 3′-hydroxylation of caffeoyl-4′-hydroxyphenyllatic acid, from *C. blumei*. Remarkably, the catalytic efficiency of the latter reaction surpasses that of the former by a factor of eight, with both catalytic processes yielding RA. Furthermore, the coexpression of the *C. blumei* cytochrome P450 reductase with *Cb*CYP98A14 amplifies the hydroxylation capacity of the enzyme sevenfold [68]. In another study, four *Sm*CYP98A enzymes have been identified in the whole genome of *S. miltiorrhiza* and shown to be involved in the biosynthesis of SAs, namely, *Sm*CYP98A75, *Sm*CYP98A76, *Sm*CYP98A77, and *Sm*CYP98A78 (*Sm*CYP98A14) [69]. Recent research has revealed that *Sm*CYP98A14 and *Sm*CYP98A75, members of the CYP98A family, are responsible for *meta*-hydroxylation reactions at the 3 and 3′ positions in the aromatic rings of RA. These two enzymes display distinct catalytic preferences for their catalytic sites, with *Sm*CYP98A75 catalysing the hydroxylation of the C-3′ position in the acyl acceptor moiety and *Sm*CYP98A14 favouring hydroxylation at the C-3 position in the acyl donor moiety of RA. Furthermore, *Sm*CYP98A75 has been identified as a key enzyme involved in the biosynthesis of DSS [38].

*Le*CYP98A6, identified from *L. erythrorhizon*, is involved in the *meta*-hydroxylation of the benzene ring in the acyl donor moiety of 4-coumaroyl-4′-hydroxyphenyllactic acid, which results in the production of caffeoyl-4′-hydroxyphenyllatic acid, the immediate precursor of RA. Moreover, the expression level of the *Le*CYP98A6 gene is positively correlated with the RA level, indicating that this gene plays a pivotal role in the RA biosynthetic pathway [59]. Other scholars have reported that *Pc*CYP98A112 from *P. campanularia* catalyzes the 3-hydroxylation of the acyl donor moiety of RA, whereas *Pc*CYP98A113 is responsible for the 3′-hydroxylation of the acyl acceptor moiety [55].

CYP98A family monooxygenases play critical roles as *meta*-hydroxylases in the biosynthesis of phenolic bioactive compounds. The overexpression of these enzymes has been successfully demonstrated to increase the accumulation of salvianolic acids in various medicinal plants. By employing metabolic engineering strategies to overexpress CYP98A, it is possible to develop medicinal plants with high levels of salvianolic acids, thereby significantly improving their medicinal quality and efficacy.

An overview of all reported CYP98A genes involved in the biosynthesis of phenylpropionic acid compounds and their associated functions is listed in Table 1.

**Table 1:** Functions of CYP98A monooxygenases in the biosynthesis of phenylpropionic acid compounds: 4-coumaroyl-4′-hydroxyphenyllactic acid (4-C-4′-HPL), 4-coumaroyl-3′,4′-dihydroxyphenyllactic acid (4-C-3′,4’-DHPL), caffeoyl-4′-hydroxyphenyllatic acid (Ca-4′-HPL), *p*-coumaroyl shikimate *(p*CSA), caffeoyl shikimate (CSA), *p*-coumaroyl quinate (*p*CQ), and chlorogenic acid (caffeoyl quinate, CGA)

**Species**	**CYP name**	**Function**	**Species**	**CYP name**	**Function**
*Arabidopsis thaliana*	*At*CYP98A3	*p*CSA→CSA	*Triticum aestivum*	*Ta*CYP98A10	*pCSA→CSA* *pCQ→CGA*
		*p*CQ→CGA			
	*At*CYP98A8	Phenol amide→ArOH			
				*Ta*CYP98A11	
	*At*CYP98A9				
				*Ta*CYP98A12	
*Brachypodium distachyon*	*Bd*CYP98A4	*p*CSA→CSA			
		*p*CQ→CGA			
*Lithospermum erythrorhizon*	*Le*CYP98A6	4-C-4'-HPL→Ca-4'-HPL	*Coleus blumei*	*Cb*CYP98A14	Ca-4'-HPL→RA
		4-C-3',4'-DHPL→RA			4-C-3',4'-DHPL→RA
*Ocimum basilicum*	*Ob*CYP98A13-1	*p*CSA→CSA	*Ruta graveolens*	*Rg*CYP98A22	*p*CSA→CSA
		*p*CQ→CGA			*p*CQ→CGA
	*Ob*CYP98A13-2		*Populus trichocarpa*	*Pt*CYP98A23	*p*CSA→CSA
*Coffea canephora*	*Cc*CYP98A35	*p*CSA→CSA		*Pt*CYP98A27	*p*CQ→CGA
		*p*CQ→CGA			
	*Cc*CYP98A36	*p*CSA→CSA		*Pt*C3H3	pCSA→CSA
*Glycine max*					
	*Gm*CYP98A2	Data not shown	*Trifolium pratense*	*Tp*CYP98A44	*p*CSA→CSA
*Podophyllum hexandrum*	*Ph*CYP98A68	Data not shown	*Cynara cardunculus*	*Cc*CYP98A49	*p*CSA→CSA
					*p*CQ→CGA
*Salvia miltiorrhiza*	*Sm*CYP98A14	4-C-4'-HPL→Ca-4'-HPL	*Lonicera japonica*	*Lj*C3'H	*p*CSA→CSA
		4-C-3',4'-DHPL→RA			
	*Sm*CYP98A75	4-C-4'-HPL→4-C-3',4'-DHPL	*Phacelia campanularia*	*Pc*CYP98A111	4-C-3',4'-DHPL→RA
		Ca-4'-HPL→RA			
	*Sm*CYP98A76	Data not shown		*Pc*CYP98A112	4-C-4'-HPL→Ca-4'-HPL
					4-C-3',4'-DHPL→RA
	*Sm*CYP98A77	Data not shown		*Pc*CYP98A113	4-C-4'-HPL→4-C-3',4'-DHPL
					Ca-4'-HPL→RA
*Leucojum aestivum*	*La*C3'H	*p*CSA→CSA	*Sorghum bicolor*	*Sb*CYP98A1	Data not shown

**Figure 5:**
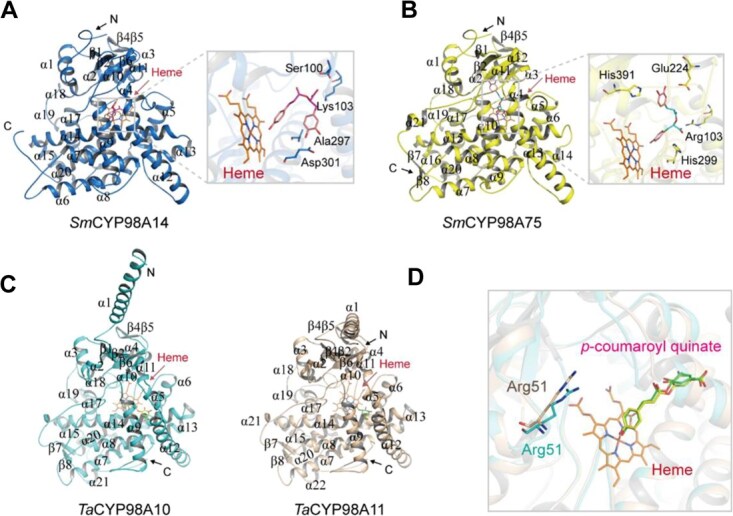
Different catalytic efficacies and substrate preferences attributed to the structural diversity of the CYP98A monooxygenases. (A) Interactions of 4-coumaroyl-3′,4′-dihydroxyphenyllactic acid and *Sm*CYP98A14 in the active pocket. (B) Interactions of caffeoyl-4′-hydroxyphenyllatic acid and *Sm*CYP98A75 in the active pocket. (C) Structures of *Ta*CYP98A10 (cyan) and *Ta*CYP98A11 (wheat) predicted by the AlphaFold server. (D) Comparison of the predicted active sites in the *Ta*CYP98A10 and *Ta*CYP98A11 models interacting with *p*-coumaroyl quinate.

The aforementioned review suggests that the CYP98A monooxygenases, which originate from angiosperms, employ *p*-coumaroyl shikimate and *p*-coumaroyl quinate as substrates in the biosynthesis of phenylpropionic acid compounds. Among some horticultural species, such as Lamiaceae and Boraginaceae, CYP98A monooxygenases play a key role in salvianolic acid biosynthesis. Some CYP98A monooxygenases possess a relatively vast substrate pool, and even within the same species, different members of the CYP98A family can have opposing substrate preferences. Substrate specificity varies depending on the species and specific subtype within the same species. These differences in catalytic efficacy and substrate preference might be attributed to variations in the enzymatic structure (Fig. 5).

### Research on the role of CYP98A in the biosynthesis of coumarin

Coumarin compounds exhibit broad-spectrum resistance against fungi, bacteria, viruses, and insects in horticultural plants [70]. Hydroxylated coumarins, represented by scopoletin and scopolin, participate in plant stress responses. Previous studies have shown that scopoletin and scopolin can effectively eliminate ROS within plants, mitigate oxidative stress responses, and stimulate the proliferation of cellular structures [71]. Scopoletin and scopolin are synthesized via the orthohydroxylation of coumaroyl-CoA by coumaroyl-CoA 2′-hydroxylase (C2′H), resulting in the formation of 2′,4′-dihydroxycoumaroyl-CoA. This compound subsequently undergoes cyclization into umbelliferone and aesculetin under the influence of COSY-mediated catalysis. Consequently, *O*-methyltransferase (OMT) catalyzes the formation of scopoletin and its glycoside scopolin. Furthermore, investigations have indicated that coumaric acid can be converted into caffeic acid by C3H. This compound can be further metamorphosed into scopoletin through the actions of catechol-*O*-methyltransferase (COMT), 4-coumarate-CoA ligase (4CL), F6′H, and COSY [72–74]. CYP98A enzymes can modulate caffeic acid production, thereby impacting the biosynthesis of scopoletin and scopolin.

Notable decreases in scopoletin and scopolin levels can be detected in the *At*CYP98A3 mutant, indicating that the biosynthesis of scopoletin and scopolin in *A. thaliana* depends on the 3′-hydroxylation of coumaric acid catalysed by *At*CYP98A3. This finding can be attributed to the mutation in *At*CYP98A3 that induces a decrease in the caffeic acid content and then sequentially reduces the accumulation of scopoletin and scopolin [75]. Cassava (*Manihot esculenta* Crantz) is a globally important food crop, and the rapid postharvest physiological deterioration (PPD) of tubers due to the accumulation of phenolic secondary metabolites after harvest reduces its palatability and marketability. The expression of the *Me*C3′H gene in transgenic cassava is inhibited via the RNAi method. Although the transgenic cassava plants maintain a normal phenotype, the levels of scopoletin and scopoline decrease, decelerating the accumulation of coumarins and extending the duration of cassava storage. These findings validate the hypothesis that the CYP98A enzyme plays a vital role in hydroxycoumarin synthesis and indicate that this enzyme is a potential target for delaying PPD symptoms in cassava through genetic engineering [76].

Scopolin and its derivative scopoletin are important natural compounds that contribute to the antimicrobial and antioxidant activities of plants. These compounds are synthesized as defence mechanisms against pathogen attacks and abiotic stresses [77–79]. Coumarins are well known in the pharmaceutical industry for their diverse therapeutic properties, making them valuable resources for drug development [80]. The biosynthesis processes of scopoletin and scopolin are dependent on the CYP98A enzyme, and this molecular understanding of coumarin biosynthesis provides valuable guidance for researchers to elucidate the physiological mechanisms of enzymes involved in coumarin production. Furthermore, leveraging metabolic engineering methods to modulate the activity of CYP98A monooxygenases is a highly promising approach for manipulating coumarin accumulation in crops. This strategy is an efficient and cost-effective technique for enhancing crop quality and resilience.

### Research on the role of CYP98A in the biosynthesis of flavonoids

In addition to being integral constituents of plant pigments, flavonoids are instrumental in numerous aspects of horticultural plant growth and development. These aspects include stress defence mechanisms, signal transduction, and antioxidant activities [81–83]. These physiological functions are indispensable for moderating the adaptation of plants to their environmental surroundings. The biosynthesis of flavonoids is the most diverse branch of the phenylpropanoid metabolic pathway. CYP98A monooxygenases are involved in phenylpropanoid metabolism, which in turn influences flavonoid biosynthesis in plants.

Research on the transcriptome of *Saposhnikovia divaricata* has revealed that the expression level of the CYP98A enzyme in roots is greater than that in leaves, with the flavonoid contents exhibiting similar patterns. Therefore, the CYP98A enzyme may be a key gene controlling the biosynthesis of flavonoid compounds in roots, indicating that the CYP98A monooxygenase is a potential candidate gene for screening the differences in flavonoid content in the roots of *S. divaricata* [84]. For apples, fruit skin colour is a key attribute of appearance and quality, and CYP98A monooxygenases can affect anthocyanin accumulation, thereby contributing to fruit colouration. The activation of CYP98A genes in apples has a considerable effect on anthocyanin accumulation during the colour transition phase, with notably increased expression levels of the CYP98A family in apples exhibiting deepened colouration [85]. In addition, the overexpression of *At*CYP98A9 suggests its involvement in the metabolism of soluble flavonoids, and *in vitro* activity assays have revealed the naringenin 3′-hydroxylase activity of *At*CYP98A9 [86].

Moreover, CYP98A monooxygenases can increase plant resistance to microorganisms via the regulation of flavonoid synthesis in plants. Verticillium wilt is a devastating disease affecting *Solanaceae* plants that results in considerable economic damage. A transcriptome analysis of *Solanum sisymbriifolium* has revealed that CYP98A monooxygenases respond to Fusarium wilt infection. This infection activates the phenylpropanoid and flavonoid biosynthetic pathways, thereby increasing plant resistance to Fusarium wilt [87]. As a highly valuable perennial medicinal herb, the roots of *Panax notoginseng* are often severely damaged by root–knot nematode (RKN) infestations. Integrated transcriptomic, metabolomic, and histochemical analyses have indicated that *P. notoginseng* may activate phenylpropanoid biosynthesis pathways by upregulating key enzymes such as CYP98A monooxygenases. This activation results in the synthesis of phenolic, flavonoid, lignin, and anthocyanin pigments, which serve as both response and defence mechanisms against RKN attacks [88].

Many types of flavonoids, such as anthocyanins, chalcones, flavonols, flavanones, flavones, and isoflavones, are commonly found in plants. Among them, anthocyanins stand out as the most potent natural water-soluble pigments within the plant kingdom. These pigments are synthesized through the phenylpropanoid branch of flavonoid biosynthesis, imparting vivid colours to horticultural plants. Furthermore, flavonoids act as effective scavengers of ROS, offer photoprotection, and function as stress signals. The activation of CYP98A monooxygenases enhances the biosynthesis of flavonoids, which not only contributes to pigmentation in flowers and fruits, protects against UV radiation, and attracts pollinators but also significantly improves the resistance of horticultural plants to various biotic and abiotic stresses. Therefore, the use of CYP98A monooxygenases can effectively increase the flavonoid content, thereby increasing the ability of horticultural plants to withstand external stresses.

### Function of CYP98A in lignin biosynthesis

Lignins, which are significant polyphenolic polymers present in plant cell walls, directly or indirectly crosslink with cellulose, hemicellulose, and additional polysaccharide molecules. Lignin plays a pivotal role in sustaining guard cell function, ensuring effective water transport, and enhancing plant resistance to biotic stress. Moreover, lignin intensifies the strength and rigidity of plant cell walls, facilitating the support of increasingly complex structures and contributing to vertical growth and morphological stability.

In the phenylpropanoid metabolic pathway, phenylalanine is transformed into *p*-coumaric acid via the activity of phenylalanine ammonia lyase (PAL) and cinnamate 4-hydroxylase (C4H). Subsequently, *p*-coumaric acid is converted into the key intermediate coumaroyl-CoA by 4CL. Following this step, catalysis by COMT and ferulate-5-hydroxylase (F5H) yields coniferaldehyde and sinapaldehyde, which are further reduced to coniferyl and sinapyl alcohol by CAD, resulting in G- and S-type lignin formation. *p*-Coumaroyl-CoA is transformed into *p*-coumaric alcohol via CCR and CAD, subsequently undergoing polymerization to produce H-type lignin [89]. These lignin monomers are interlinked through radical polymerization to form lignins. CYP98A monooxygenases catalyse the *meta*-hydroxylation of cinnamic acid and *p*-coumaroyl-CoA, leading to the production of caffeic acid and caffeoyl-CoA, which contribute to lignin biosynthesis.

Compared with the wild-type strain, the *At*CYP98A3 mutant *REF8* presents significant developmental defects and a diminished lignin content in the stems. Moreover, abnormal lignification clearly occurs within the roots and is characterized predominantly by the deposition of H-lignin, which is principally formed from the polymerization of *p*-coumaric alcohol. H-lignin is a minor component of lignins in angiosperms, and the increase in H-lignin content in *REF8* may be attributed to mutations in CYP98A, which effectively block the metabolic flow of lignin biosynthesis from caffeoyl-CoA biosynthesis. This obstruction inevitably inhibits the formation of G-lignin and S-lignin, subsequently leading to an accumulation of H-lignin. This change in lignin content results in developmental defects and increases the susceptibility of plants to fungal attack, indicating that phenylpropanoid pathway products catalysed by the CYP98A monooxygenase are essential for normal plant development and disease resistance [44]. Furthermore, the *At*CYP98A3 mutant obtained through T-DNA insertion exhibits a more complete loss of function than *REF8*. Owing to the reduced lignin levels, this mutant presents more stunted growth than the wild-type plants. A plausible explanation for this phenomenon is that a decrease in the lignin content triggers the collapse of the vascular conduits of the plant [90].

Lignin is an essential heterogeneous structural polymer of monolignols that forms during secondary growth and provides rigidity and strength to the plant cell wall. This polymer enhances the mechanical strength and hydrophobicity of the plant cell wall, supporting the growth of aboveground parts and water transport. Therefore, lignin metabolism plays a key role in horticultural plant defence mechanisms against stresses. Mutation of *At*CYP98A3 in Arabidopsis demonstrates that CYP98A is a key enzyme involved in phenylpropanoid and lignin metabolism and that manipulation of the expression of the CYP98A gene is a useful approach for manipulating phenylpropanoid metabolism to benefit agriculture and forestry.

## Research on the structures of plant CYP monooxygenases

The substrate selectivity levels of proteins are determined primarily by the fundamental active sites embedded in their structures, which are conventionally composed of distinct amino acid residues. These residues form a three-dimensional configuration that enables the protein to bind specific substrate molecules effectively. Most studies concerning protein–substrate binding and drug–target interactions are predicated on this principle. However, the paucity, instability, and vulnerability to external disruptions of plant-derived CYP enzymes pose formidable challenges to structural studies of these proteins in the research domain [91].

The first crystal structure of a CYP enzyme was derived from *Pseudomonas putida* P450cam, which was deciphered by Poulos in 1985, achieving a resolution of 2.6 Å [92]. Coinciding with advancements in contemporary biotechnology and structural biology, an increasing number of CYP enzyme crystal structures have been discovered, facilitating more profound explorations into the structure–function–mechanism correlations of P450 enzymes. To date, the crystal structures of five plant-derived CYP enzymes have been determined: *Sm*CYP76AH1 [93] (complex crystal structure with the P450 enzyme inhibitor 4-phenylimidazole), *At*CYP97A3 [94] (complex formed without substrate and with non-natural substrates), *At*CYP97C1 [94] (detergent-bound form), *Sb*C4H1 [95], and *At*CYP90B1 [96] (complex crystal structure with the substrate cholesterol). The three-dimensional structure of the critical catalytic enzyme *Sm*CYP76AH1, which plays a pivotal role in the biosynthetic pathway of the significant active compound tanshinone in *S. miltiorrhiza*, and the complex crystal structure orchestrated with the CYP enzyme inhibitor 4-phenylimidazole were resolved at a resolution of 2.6 Å [93]. Furthermore, the indispensable amino acid residues associated with the catalytic function of this structure have been elucidated via X-ray diffraction methods. Although it has considerable potential for resolution enhancement, the disclosed crystal structure is the first reported for a CYP involved in the secondary mechanism of medicinal plants. This structure offers a more accurate model reference for homologous modelling experiments of functional plant genes. This approach holds considerable importance for the molecular docking of protein active sites and for the prediction of protein functions.

A comparison of the three-dimensional structures of CYPs derived from different sources has revealed that the spatial structure of CYP protein typically exhibits an inverted triangular topology. The core architecture is a trigonal prism consisting of four α-helices: D-, E-, I-, and L-helices. Substrate entry into the CYP active site is correlated with the structural flexibility of the B- and C-loops and the F- and G-helices. The conformational adaptations in the F- and G-helices facilitate seamless substrate ingress into the active site of the enzyme and promote the efficient egress of associated products. Although the amino acid sequence similarity levels among different CYP enzymes can be as low as 20%, their three-dimensional structures are notably conserved, particularly in the I- and L-helices proximate to the haem iron. The amino acid residues within the I-helix primarily participate in the activation of oxygen molecules during the catalytic process, whereas a cysteine residue located at the N-terminus of the L-helix is entirely conserved and tethered to the iron atom in the haem iron via a thiol bond. However, the substrate-binding sites of CYPs lack such conservation; that is, the amino acid residues in the substrate recognition sites of disparate CYPs may significantly vary. This variability underpins the structural basis for the functional diversity of this enzyme family.

**Figure 6:**
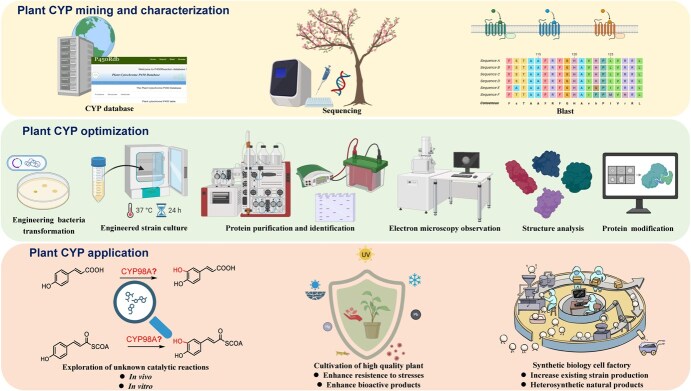
Summary of the research strategies and applications in plant monooxygenase studies. Image created with BioRender.com with permission.

## Summary and prospects of CYP and CYP98A studies

Phenolic compounds play critical roles in plant physiology, contributing not only to the stress resistance capabilities of plants but also to their growth, development, and structural advancement. Consequently, these compounds have remained a significant focus of scientific research. CYP98A enzymes are involved in the *meta*-hydroxylation of phenylpropanoid compounds, which are common precursors of phenolic compounds, thereby influencing the direction of metabolic flow.

To date, many researchers investigating CYP98A have focused primarily on the transition of coumaric acid derivatives into caffeic acid derivatives, with a specific emphasis on the *meta*-hydroxylation of 4-hydroxycinnamic acid esters. Nevertheless, controversy persists regarding whether coumaric acid or coumaroyl-CoA can be catalysed to produce caffeic acid or caffeoyl-CoA under the action of CYP98A enzymes. Recent research has shown that the direct catalysis of coumaric acid into caffeic acid by CYP98A presents challenges due to its slow reaction rates, low yields, and difficult-to-detect products [35]. Additionally, the reactions facilitated by APX/C3H lack further empirical substantiation [65–67]. Moreover, no research has reported the conversion of *p*-coumaroyl-CoA to caffeoyl-CoA by CYP98A. Determining whether the aforementioned reactions are mediated by CYP98A monooxygenases is highly important for the analysis of phenolic compound biosynthesis and metabolic regulation. This knowledge may improve our understanding of the phenylpropanoid metabolic pathway.

Research concerning CYP98A has focused primarily on the functional characterization of *meta*-hydroxylation. While the spatial and chemical selectivities of these oxidases for different substrates have been determined, no reports have provided structural bases for both their selectivities and catalytic function. Currently, the published structures of plant-derived CYP proteins are limited, possibly due to the primary location of CYPs on membrane structures, such as the endoplasmic reticulum, and their low expression levels, which render the acquisition of protein crystals challenging. Up to now, investigations of the structures of the CYP98A family of oxidases have been largely conducted via homology modelling and molecular docking [38, 55]. Although this computer-assisted structural research technique offers researchers an approximate understanding of protein information, its accuracy can be further enhanced, and it is limited by various factors, such as limitations in model construction, protein dynamics, and discrepancies between experimental and computational conditions. This accuracy gap is conspicuous compared with the results obtained from actual protein purification and cocrystallization with substrates.

Hence, employing structural biology to analyse the structures of CYP98A proteins will be a focal point in future research on CYP98A monooxygenases. We propose that structural biology studies on CYP98A should be focused on structural resolution, catalytic mechanism investigations, and enzyme engineering on the basis of relevant structural information. Specifically, the first step involves X-ray crystallography or cryo-electron microscopy to determine the crystal structure of the target CYP98A family monooxygenase, clarifying the detailed conformations of its active centre, substrate-binding pocket, and key catalytic residues. Furthermore, by integrating molecular dynamics simulations and quantum chemical calculations, the detailed mechanism of the *meta*-hydroxylation reaction catalysed by the target CYP98A family monooxygenase can be evaluated, including the key steps underlying the process, such as oxygen activation, substrate binding, and hydroxyl transfer. Finally, on the basis of structural information, the CYP98A family of monooxygenases should be rationally designed and modified. By mutating key amino acid sites and reshaping the conformation of the substrate-binding pocket to accommodate substrates with different structures, their catalytic efficiency and substrate selectivity can be optimized. This optimization should lead to the development of highly efficient *meta*-hydroxylases. In future endeavours, researchers can utilize synthetic biology to create engineered bacterial or yeast strains with high yields of phenolic compounds. Optimized CYP98A enzymes can also be introduced into plants for artificial selection and directional breeding, effectively increasing the content of active phenolic compounds. This modification not only helps plants improve their resistance to both biotic and abiotic stresses but also contributes to the breeding of high-quality plants with increased phenolic bioactive compound accumulation, thereby advancing the sustainable utilization and development of plant resources.

Based on current research on CYP98A enzymes, we summarize a research strategy involving gene mining, structural optimization, and application in the study of plant CYP monooxygenases (Fig. 6). By utilizing the P450 database and advancements in sequencing technologies, target P450 genes can be mined and characterized through sequence information. Molecular biology methods can be employed to identify the structures of P450 enzymes. Additionally, we can optimize their structures to achieve ideal catalytic characteristics through computational biology, thereby meeting our needs. Engineered P450 enzymes with enhanced catalytic efficiency and substrate selectivity can be developed through protein structure modification. This approach will aid in identifying unknown reactions catalysed by P450 enzymes, providing potential targets for regulating secondary metabolism to cultivate high-quality plants that can withstand stress and produce relatively high levels of bioactive compounds. Moreover, this research can promote the development of synthetic methods for increasing the production of natural compounds.

## Data Availability

Data sharing is not applicable to this article as no new data were created or analyzed in this study.
